# The Relationship between Intracarotid Plaque Neovascularization and Lp (a) and Lp-PLA2 in Elderly Patients with Carotid Plaque Stenosis

**DOI:** 10.1155/2022/6154675

**Published:** 2022-04-21

**Authors:** Chang Sun, Na Xi, Zhijun Sun, Xinxin Zhang, Xiaowei Wang, Huiyang Cao, Xiaowei Jia

**Affiliations:** ^1^Cadre Diagnosis and Treatment Department, Eighth Medical Center of PLA General Hospital, Beijing, China; ^2^Department of Pharmacy, Medical Supplies Center of Chinese PLA General Hospital, Beijing, China; ^3^Department of Cardiovascular, The First Medical Center of PLA General Hospital, Beijing, China; ^4^School of Foreign Languages English, Guilin University of electronic technology, Guilin, China; ^5^Personnel Section, Liaoning Fuxin Center Hospital, Fuxin, China; ^6^Cardiac Function Division, Liaoning Fuxin Center Hospital, Fuxin, China

## Abstract

The aim of this study was to investigate the relationship between carotid plaque neovascularization and lipoprotein (a) [Lp (a)], lipoprotein-associated phospholipase A2 (Lp-PLA2) in elderly patients with carotid plaque stenosis. One hundred elderly patients with carotid plaque stenosis diagnosed in our hospital from January 2020 to January 2022 were retrospectively analyzed and divided into stable (*n* = 62) and unstable (*n* = 38) groups according to whether the plaque was stable or not. Plasma Lp (a), Lp-PLA2, apoA, and apoB levels were measured; intraplaque angiogenesis (IPN) scores were examined by contrast-enhanced ultrasound (CEUS) to assess IPN grade in patients; and Pearson correlation was used to analyze the relationship between plasma Lp (a) and Lp-PLA2 levels and plaque characteristics and angiogenesis. The maximum thickness and total thickness of carotid plaque in the unstable group were significantly greater than those in the stable group (*P* < 0.05); the IPN grade was mainly grade III and IV in the unstable group and grade II in the stable group, and the IPN score was significantly higher in the unstable group than in the stable group (*P* < 0.05); there was no significant difference in the plasma apoA and apoB levels between the two groups (*P* > 0.05), and the plasma Lp (a) and Lp-PLA2 levels were significantly higher in the unstable group than in the stable group (*P* < 0.05); the neovascular grade, plasma Lp-PLA2, and Lp (a) levels were significantly increased (*P* < 0.05); the plasma Lp (a) and Lp-PLA2 levels were positively correlated with the maximum plaque thickness, total plaque thickness, degree of stenosis, and angiogenesis (*P* < 0.05). The plasma levels of Lp (a) and Lp-PLA2 are positively correlated with intraplaque angiogenesis, and their levels can reflect the stability of carotid plaques.

## 1. Introduction

Carotid plaque is usually caused by lipid deposition at the bifurcation of common carotid artery, without obvious symptoms at the early stage. Severe stenosis may be accompanied by dizziness, headache, and fainting, which is a common cause of stroke and acute cerebral infarction. The incidence of carotid plaque increases with age, the intima media thickens, and carotid plaque and carotid stenosis or occlusion generally develops in a progressive manner [1, 2]. With the development of medical imaging technology, carotid artery stenosis and plaque formation can be diagnosed and assessed clinically by CT, MRI, carotid ultrasound, and carotid angiography [3]. Current studies have found that intraplaque neovascularization (IPN) is an important predictor of plaque hemorrhage and rupture [4]. Contrast-enhanced ultrasound (CEUS) technique is a means of accurate clinical detection of IPN. After injection of contrast microbubbles, the movement direction of contrast microbubbles into the plaque is identified. When the contrast microbubbles move from the adventitial side of the plaque or the shoulder side of the plaque to the core side of the plaque, it indicates the presence of IPN [5]. Lipoprotein (a) [lipoprotein (a), Lp (a)] is a low-density lipoprotein whose level is mainly genetically determined and consists of apolipoprotein A (apoA) covalently linked to apolipoprotein B-100 (apoB) [6]. It has been suggested that Lp (a) is involved in the development of atherosclerosis, and plasma Lp (a) levels are closely related to thrombosis and damage [7]. It has also been suggested that Lp (a) does not promote or inhibit neovascularization [8]. Whether plasma Lp (a) levels have an effect on IPN, resulting in plaque instability, remains inconclusive. Lipoprotein-associated phospholipase A2 (Lp-PLA2) is a novel vessel-specific inflammatory marker mainly secreted by macrophages and lymphocytes in atherosclerotic plaques [9]. Studies have found that intraplaque neovascularization is an important indicator of plaque stability, which can deposit a large number of inflammatory matrix in plaques, promote lymphocyte accumulation, and aggravate plaque instability [10]. The aim of this study was to analyze the relationship between carotid intraplaque angiogenesis and Lp (a) and Lp-PLA2 in elderly patients with carotid artery stenosis and to investigate the significance of plasma Lp (a) and Lp-PLA2 levels in predicting plaque stability and intraplaque angiogenesis.

## 2. Materials and Methods

### 2.1. Clinical Data

This study was approved by the Ethics Committee of Eighth Medical Center of PLA General Hospital. Signed written informed consents were obtained from all participants before the study. A total of 100 elderly patients with carotid plaque stenosis who were diagnosed in our hospital from January 2020 to January 2022 were retrospectively analyzed and divided into stable group (*n* = 62) and unstable group (*n* = 38) according to whether the plaque was stable or not. Inclusion criteria were shown as follows: (1) Carotid artery stenosis was diagnosed by carotid artery color ultrasound and other imaging examinations. (2) Patients' age was ≥60 years old. (3) Patients can tolerate CEUS examination. Exclusion criteria were shown as follows: (1) Patients were allergic to contrast agent. (2) Patients were neurological dysfunction or cognitive deficiency. (3) Patients were combined with severe infection. (4) Patients were combined with obvious liver, kidney, and other vital organ dysfunction.

### 2.2. Outcome Measures and Test Methods

The admission cases were retrospectively analyzed, and general data such as gender, age, disease history, drug treatment history, and plaque status of the patients were recorded.

### 2.3. Carotid CEUS Examination

All patients who underwent CEUS had signed the angiographic informed consent.

GE Vivid E95 ultrasonic diagnostic apparatus, high-frequency superficial probe, and contrast mode were used. A 1.5 mL Sono Vue microbubble suspension (Bracco, Italy) was bolus injected through the superficial vein of the elbow, followed by 2~3 mL normal saline at the same rate to observe the contrast agent development effect in the carotid artery lumen, and DICOM format images 3 s before and 5 min after the appearance of contrast agent in the carotid artery lumen were collected and stored for real-time dynamic analysis. On CEUS examination, no microbubbles seen in the plaque were defined as an IPN score of 0; microbubbles confined to the shoulder or adventitial side of the plaque were defined as an IPN score of 1; and microbubbles seen throughout the plaque were defined as an IPN score of 2. A sum of IPN scores ≥2 was defined as unstable plaque, and a sum of IPN scores <2 was defined as stable plaque.

Grading criteria for intraplaque neovascularization [11, 12] are as follows: It is divided into 4 grades according to the degree of signal enhancement in several plaques, namely, grade I, no enhancement of plaques; grade II, punctate enhancement in or around plaques; grade III, scattered punctate and linear enhancement in and around plaques; and grade IV, diffuse punctate and linear enhancement in and around plaques.

### 2.4. Plasma Lipoprotein and Lp-PLA2 Levels

4 mL venous blood was collected from all patients in the morning and placed in an anticoagulant tube containing sodium citrate, mixed well, and centrifuged at 3000 r/min for 5 min at 5°C, and the plasma was separated and stored in a −20°C freezer for testing.

In determining the plasma Lp-PLA2 levels, plasma Lp-PLA2 levels were measured by immunoturbidimetric assay. The reagents were produced and provided by Wuhan Huamei Biological Engineering Co., Ltd. (Wuhan, China), and the operation was performed in strict accordance with the kit instructions.

In determining the plasma lipoprotein, the plasma levels of apolipoprotein A (apoA) and apolipoprotein B (apoB) were measured by Hitachi 7060 automatic biochemical analyzer, and the plasma Lp (a) level was measured by immunoturbidimetry. The reagents were produced and provided by Wuhan Huamei Biological Engineering Co., Ltd. (Wuhan, China). The operation process was performed in strict accordance with the instructions on the kit.

### 2.5. Statistical Analysis

Statistical Product and Service Solutions (SPSS) 21.0 software (IBM, Armonk, NY, USA) was used to statistically analyze the obtained data. The measurement data satisfying normal distribution were expressed as (−*x* ± *s*), and the two-sample independent *t*-test was used to compare the group differences between patients in the stable group and those in the unstable group; one-way ANOVA was used for the comparison between three groups and above; the count data were expressed as the number of cases (*n*) or rate (%); and the *χ*^2^ test was used to compare patients in the stable group and those in the unstable group, differences between groups of patients, and Pearson correlation analysis of plasma Lp (a) and Lp-PLA2 levels in relation to plaque characteristics and revascularization; *P* < 0.05 indicates a statistically significant difference.

## 3. Results

### 3.1. Baseline Data of Patients in Stable Group

There was no significant difference in age, gender, disease history, drug treatment history, degree of carotid artery stenosis, reasons for treatment, and other clinical data between the stable group and the unstable group (*P* > 0.05); the maximum thickness and total thickness of carotid plaques in the unstable group were significantly greater than those in the stable group (*P* < 0.05) (see Table 1).

### 3.2. IPN Score and IPN Imaging Grade in Patients with Stable and Unstable Disease

The IPN grade was mainly grade III and IV in the unstable group and grade II in the stable group (*P* < 0.05); the IPN score was significantly higher in the unstable group than in the stable group (*P* < 0.05) (see Table 2).

### 3.3. Plasma Lipoprotein and Lp-PLA2 Levels in Patients with Stable and Unstable Disease

There is no significant difference in plasma apoA and apoB levels between the stable and unstable groups (*P* > 0.05); plasma Lp (a) and Lp-PLA2 levels are significantly higher in the unstable group than in the stable group (*P* < 0.05), as shown in Table 3. The plasma levels of Lp-PLA2 and Lp (a) are significantly increased (*P* < 0.05), as shown in Table 4.

### 3.4. Correlation of Plasma Lp (a) and Lp-PLA2 Levels with Plaque Characteristics and Angiogenesis

Variable assignment was performed for the degree of plaque stenosis, mild =1, moderate =2, severe = 3, and occlusion = 4; Pearson correlation analysis results showed that plasma Lp (a) and Lp-PLA2 levels were positively correlated with the maximum plaque thickness, total plaque thickness, degree of stenosis, and angiogenesis, that is, plasma Lp (a) and Lp-PLA2 levels; and the more narrow the plaque thickness, the more likely it was to have plaque angiogenesis (*P* < 0.05) (see Table 5).

## 4. Discussion

Atherosclerosis is a common type of cardiovascular disease in the elderly population. Carotid atherosclerotic plaque is a natural part of the atherosclerosis process and tends to occur at the bifurcation of the carotid artery and at the beginning of the segment where blood flow is slower [13, 14]. The higher IPN classification suggests a greater risk of plaque instability, plaque dislodgement, or even intraplaque hemorrhage [15]. Since the formation of atherosclerotic plaques is associated with abnormal lipid levels and their stability is related to revascularization, this study aims to establish a link between lipid levels and plaque stability by investigating the relationship between lipid-related indicators and revascularization.

The results of this study showed that the maximum and total thickness of carotid plaque was significantly greater in the plaque instability group than in the stable group and that plaque thickness was associated with carotid stenosis, with thicker plaque thickness indicating greater stenosis in the carotid artery. The higher the plaque instability, the higher the likelihood of its detachment from the carotid artery wall, and the higher the likelihood of blockage of other vessels.

Plaque instability is high, the possibility of detachment from the carotid artery wall, and the greater the possibility of other vascular blockages. The results of CEUS examination showed that the IPN grade was mainly grade III and IV in the unstable group and grade II in the stable group, and the IPN score was significantly higher in the unstable group than in the stable group, indicating that more intraplaque angiogenesis was observed in the unstable group. Previous studies have suggested [16, 17] that the degree of carotid artery stenosis is related to the biological characteristics of plaques, and patients in the unstable group have a high IPN grade, which may indicate the degree of carotid plaque stenosis in patients in the unstable group. Plaque stability significantly affects the occurrence of ischemic stroke and other diseases. Due to plaque instability, there is a risk of detachment and rupture at any time. When plaque ruptures and detaches, it falls off from the vessel wall and moves along the blood circulation, resulting in vascular embolism and inducing stroke [18].

The results of this study showed that plasma Lp (a) and Lp-PLA2 levels were significantly higher in the unstable group than in the stable group, and the higher the neovascular grading, the significantly higher the plasma Lp-PLA2 and Lp (a) levels. Lp (a) is a special macromolecular lipoprotein whose main physiological function is to prevent intravascular thrombolysis and pathologically promote atherogenesis, and its plasma level is mainly regulated by genes [19]. Lp (a) levels have a skewed distribution in the population with large individual differences, but the levels are basically stable in the same body and should be concerned when they show pathological increases [20]. Previous studies have shown that Lp (a) is an independent risk factor for diseases such as stroke and coronary heart disease [21]. Plasma Lp (a) levels in patients with instability indicate that high plasma Lp (a) levels indicate plaque instability, while high plaque instability also indicates more plaque angiogenesis, which may be related to the prothrombotic effect of Lp (a), and high levels of Lp (a) promote plaque instability. Previous studies have also confirmed that plaque progression and stability are closely related to IPN, because IPN increases the possibility of plaque rupture and shedding, and vessels are more likely to embolize [22]. Therefore, patients with high plasma Lp (a) levels have increased intraplaque angiogenesis, but also increase plaque instability, resulting in their rupture and shedding, ischemic stroke, and other serious events. Under physiological conditions, hemoglobin in the arterial intima provides nutrition for it. After carotid artery stenosis occurs in patients, the intima media of blood vessels thickens, resulting in local tissue hypoxia and inflammation. Under these stimuli, vascular endothelial growth factor is highly expressed, so new blood vessels are formed [23, 24]. The plasma Lp-PLA2 level can reflect the level of inflammation, which is mainly secreted by macrophages and lymphocytes in atherosclerotic plaques, and its level indicates the level of arterial plaque inflammation and increased plaque angiogenesis [25]. Plaque angiogenesis is one of the evaluation criteria for plaque stability. The degree of angiogenesis in the plaque instability group is higher than that in the stable group. Patients in the plaque instability group had a higher degree of angiogenesis classification than those in the stability group, and their plasma Lp-PLA2 levels were also higher, indicating that patients in the plaque instability group also had significantly higher levels of inflammation than those in the instability group, establishing a link between plasma Lp-PLA2 levels, plaque stability, plaque angiogenesis, and plaque inflammation levels.

The results of Pearson correlation analysis showed that the plasma levels of Lp (a) and Lp-PLA2 were positively correlated with the maximum plaque thickness, total plaque thickness, and degree of stenosis and angiogenesis, that is, the plasma levels of Lp (a) and Lp-PLA2. The more narrow the plaque thickness, the more likely it was to appear plaque angiogenesis, indicating that the plasma levels of Lp-PLA2 and Lp (a) have certain value for predicting the occurrence of IPN and plaque progression.

In summary, plasma Lp (a) and Lp-PLA2 levels are associated with carotid plaque characteristics and angiogenesis in patients with carotid artery stenosis, and the plaque stability and the related disease progression can be assessed clinically by detecting plasma Lp (a) and Lp-PLA2 levels. The shortcomings of this study are that the limited number of samples, limited to single-center studies, may produce some statistical error on the results, and retrospective analysis cannot draw causal inferences. In addition, plasma Lp (a) levels are regulated by genes, and external factors such as lifestyle habits have little effect on them, so the effect of genetic variation on plasma Lp (a) levels should also be considered. However, this study still established a link between plasma Lp (a) and Lp-PLA2 levels and plaque angiogenesis.

Plasma Lp (a) and Lp-PLA2 levels correlated well with the characteristics of carotid plaques in patients with carotid stenosis and have been reported in several publications. Plasma Lp (a) and Lp-PLA2 can reflect the stability of plaque and may be clinically useful in the treatment and prognosis of patients.

## Figures and Tables

**Table 1:** Baseline data of patients in stable group (*n*, *x̅*±*s*).

Metrics	Stable group (*n* = 62)	Unstable group (*n* = 38)	*Χ* ^2^/*t*	*P*
Age (years)	70.67 ± 8.25	71.28 ± 8.14	0.361	0.719
Sex			0.075	0.784
Male	36	23		
Female	26	15		
Hypertension			0.214	0.643
Yes	43	28		
No	19	10		
Diabetes			1.831	0.176
Yes	15	14		
No	47	24		
Cerebral infarction			3.265	0.071
Yes	14	15		
No	48	23		
Coronary heart disease			3.552	0.059
Yes	28	10		
No	34	28		
Statin therapy			1.045	0.307
Yes	55	36		
No	7	2		
Degree of carotid artery stenosis			3.026	0.388
Mild	25	13		
Moderate	12	5		
Severe	18	11		
Occlusion	7	9		
Reason for visit			2.464	0.292
Coronary ischemia	31	25		
Cerebral artery ischemia	20	9		
Other	11	4		
Maximum thickness of carotid plaque (mm)	3.82 ± 1.16	4.37 ± 1.25	2.234	0.028
Total thickness of carotid plaque (mm)	6.21 ± 2.14	7.19 ± 2.06	2.254	0.026

**Table 2:** IPN score and IPN development grade in stable patients (*n*, *x̅*±*s*).

Metrics	Stable group (*n* = 62)	Unstable group (*n* = 38)	*Χ* ^2^/*t*	*P*
Neovascularization (case)			11.963	0.008
Grade I	16	4		
Grade II	27	9		
Grade III	11	14		
Grade IV	8	11		
IPN score (points)	1.05 ± 0.33	2.17 ± 0.62	11.782	< 0.001

**Table 3:** Plasma lipoprotein and Lp-PLA2 levels in patients with stable disease (*x̅*±*s*).

Metrics	Stable group (*n* = 62)	Unstable group (*n* = 38)	*t*	*P*
ApoA (mmol/L)	1.17 ± 0.15	1.13 ± 0.14	1.327	0.188
ApoB (mmol/L)	0.85 ± 0.11	0.81 ± 0.12	1.705	0.091
Lp (a) (mg/L)	162.29 ± 51.56	248.74 ± 106.31	5.453	< 0.001
Lp-PLA2 (ng/mL)	213.86 ± 78.69	297.32 ± 91.27	4.842	< 0.001

**Table 4:** Relationship between plasma Lp-PLA2 and Lp (a) levels and neovascularization grade (*x̅*±*s*).

Metrics	Number of subjects	Lp (a) (mg/L)	Lp-PLA2 (ng/mL)
Neovascularization		195.14	245.57
Grade I	20	151.38 ± 45.94	199.56 ± 71.13
Grade II	36	181.91 ± 62.27	223.70 ± 79.85
Grade III	25	204.43 ± 88.17	271.71 ± 83.84
Grade IV	19	254.05 ± 109.63	301.05 ± 92.29
F		6.231	6.742
*P*		< 0.001	< 0.001

**Table 5:** Correlation of plasma Lp (a) and Lp-PLA2 levels with plaque characteristics and IPN.

Metrics	Lp (a)			Lp-PLA2		
	*R*	95% CI	*P*	*R*	95% CI	*P*
Maximum thickness of plaque	0.290	0.099 to 0.460	0.003	0.204	0.008 to 0.385	0.042
Total plaque thickness	0.235	0.040~ 0.412	0.019	0.342	0.156 to 0.505	< 0.001
Extent of stenosis	0.336	0.149 to 0.499	< 0.001	0.431	0.256~0.578	< 0.001
IPN score	0.437	0.263 to 0.584	< 0.001	0.450	0.278 to 0.594	< 0.001

## Data Availability

The datasets used and analyzed during the current study are available from the corresponding author on reasonable request.
